# Supplementary material for: COVID-19 Incidence Proportion as a Function of Regional Testing Strategy, Vaccination Coverage, and Vaccine Type
Source: Viruses. 2023 Oct 30;15(11):2181. doi: 10.3390/v15112181 (PMC10675075; doi:10.3390/v15112181)
Supplement: Supplementary file 1 [file viruses-15-02181-s001.zip › viruses-2654425-supplementary Figures.pdf]

Supplementary Figures

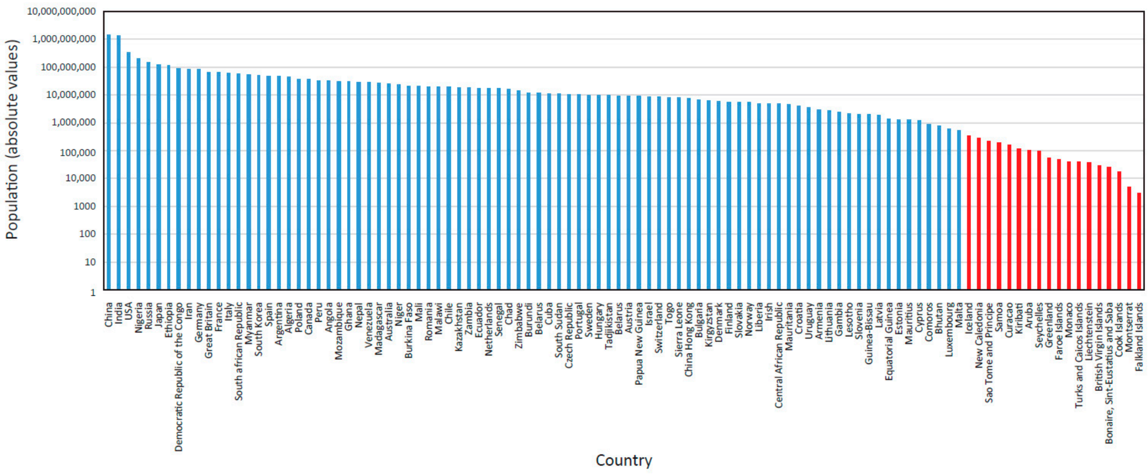

Figure S1. Distribution of countries (n=104) included in the original data set (Table S1) by population

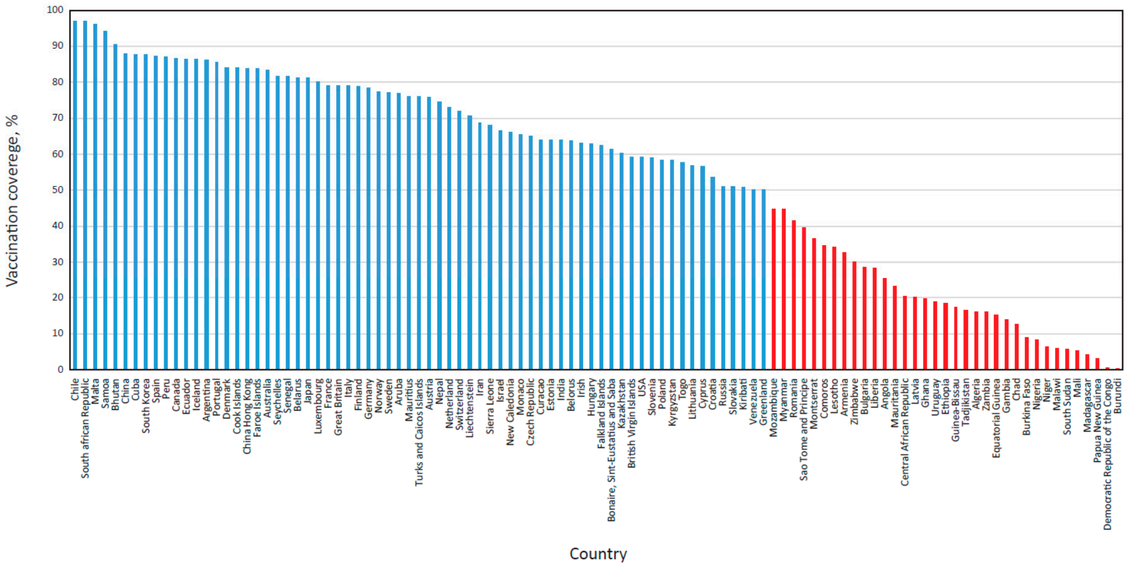

Figure S2. Distribution of countries (n=104) included in the original data set (Table S1) by vaccination coverage (%)

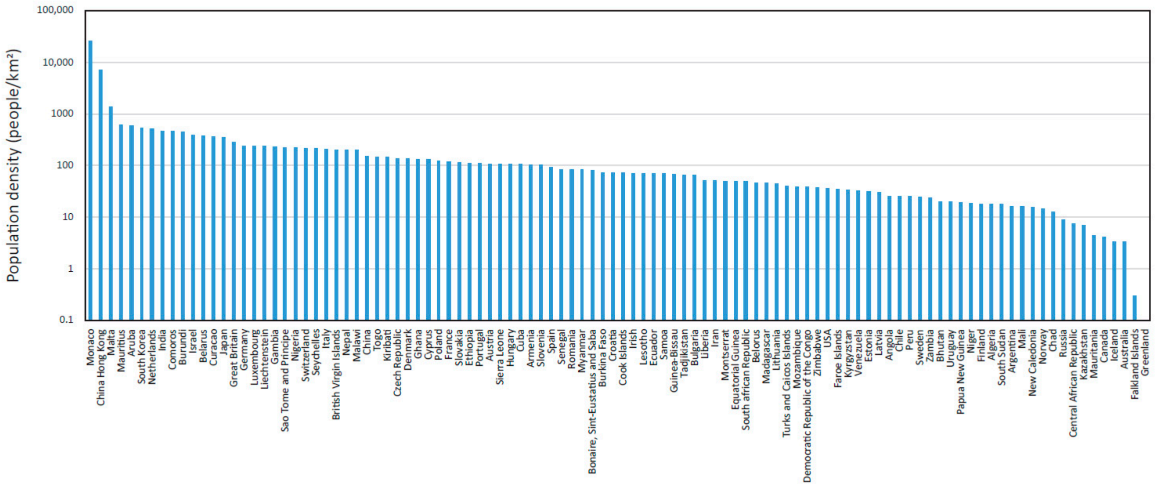

Figure S3: Distribution of countries (n=104) included in the original data set (Table S1) by population density (per km2)

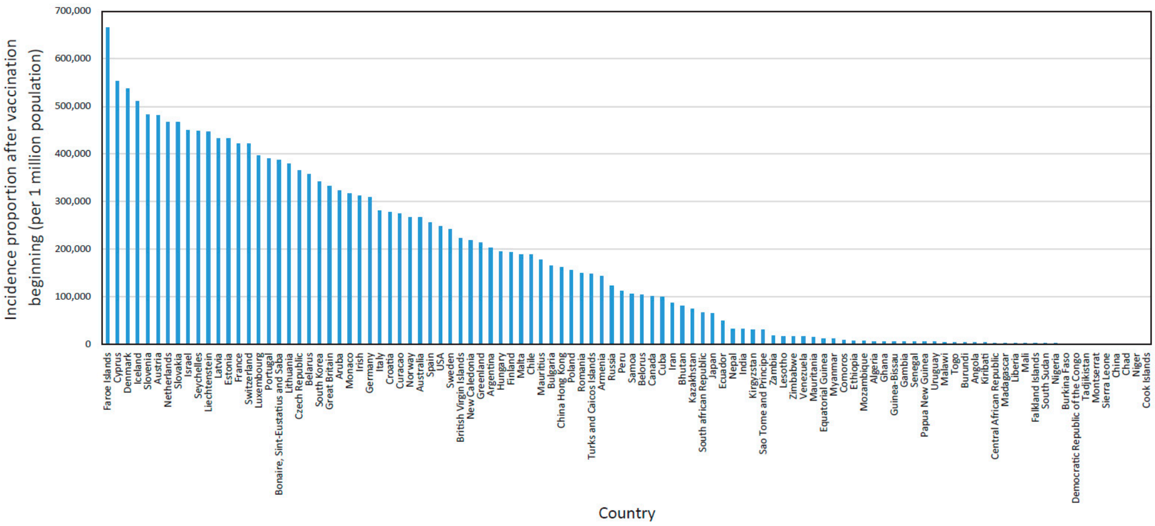

Figure S4: Distribution of countries (n=104) included in the original data set (Table S1) by COVID-19 incidence proportion (cumulative confirmed cases per 1 million population) after beginning of national vaccination programs.
