# Supplementary material for: COVID-19 Incidence Proportion as a Function of Regional Testing Strategy, Vaccination Coverage, and Vaccine Type
Source: Viruses. 2023 Oct 30;15(11):2181. doi: 10.3390/v15112181 (PMC10675075; doi:10.3390/v15112181)
Supplement: Supplementary file 1 [file viruses-15-02181-s001.zip › viruses-2654425-supplementary Tables.pdf]

Supplementary Materials

Table S1. Initial data set (n=104).

Country	Population	Population density. per km^2	PCR testing coverage, ‱	Reported COVID-19 cases, per 1 million population	Vaccination coverage. %	mRNA Vaccines (V1), %		Vector Vaccines (V2), %				Protein, Peptide Vaccines (V3), %	Whole-virion, Inactivated Vaccines (V4), %	Beginning of national vaccination company	Reported COVID-19 cases, per 1 million population before beginning of vaccination	Reported COVID-19 cases, Reported COVID-19 cases, per 1 million population after beginning of vaccination
						Pfizer / BioNTech	Moderna	Johnson & Johnson	Oxford / AstraZeneca, Covishield	Sputnik V (Gam-COVID-Vac)	CanSino					
Australia	25500000	3.3	2755	266595	83.29	55.37	0	0	44.63	0	0	0	0	21/02/2021	1105	265490
Austria	9006000	108.7	20551	480347	75.8	80.65	8.68	1.99	8.63	0	0	Novavax – 0.05	0	08/01/2021	41364	438983
Algeria	43851000	18.1	n/d	6183	16.0	0	0	0	Oxford / AstraZeneca	Sputnik V	0	0	Sinovac Sinopharm	29/01/2021	2369	3814
Angola	32866000	25.5	47	3197	25.3	0	0	0	100	0	0	0	0	01/03/2021	585	2612
Argentina	46403000	16.4	791	202500	86.1	17.16	7.62	0	26.08	20.19	0.78	0	Sinopharm – 28.16	29/12/2020	36361	166139
Armenia	2963000	103.9	1040	143006	32.6	0.8	16.1	0	18.8	16.3	0	0	Sinopharm – 43.3 CoronaVac – 4.7	31/03/2021	69283	73723
Aruba	107000	590.6	n/d	323358	77.0	100	0	0	0	0	0	0	0	29/03/2021	85225	238133
Belarus	9449000	46.6	1400	104411	63.8	0	0	0	0	57.4	0	EpiVacCorona – 0.3	Sinovac – 0.6 Sinopharm – 32.3 CoviVac – 0.5	28/12/2020	19778	84633
Belgium	11590000	381.1	2920	357100	81.23	70.15	16.92	1.69	11.24	0	0	Novavax – <0.01	0	28/12/2020	54940	302160
Bulgaria	6948000	64.5	1442	165900	28.7	65.99	11.31	11.81	10.89	0	0	0	0	29/12/2020	29105	136795
Caribbean Netherlands	26000	79.2	n/d	386501	61.4	100		0	0	0	0	0	0	09/04/2021	54635	331866
British Virgin Islands	30000	200.2	n/d	222777	59.2	0	0	100		0	0	0	0	21/05/2021	7915	214862
Burkina Faso	20903000	74.3	n/d	1001	8.89	0	0	Johnson & Johnson	Oxford / AstraZeneca	0	0	0	Sinopharm	02/06/2021	593	408
Burundi	11891000	449	127	3821	0.1	0	0	Johnson & Johnson		0	0	0	Sinopharm	19/10/2021	1298	2523
Bhutan #	772000	20	2170	80378	90.46	0	50	0	50	0	0	0	0	26/03/2021	1113	79265
Great Britain	67886000	279.1	7348	331660	79.0	48.7	3	0	48.3	0	0	0	0	10/01/2021	46584	285076
Hungary	9660000	107	1136	195400	62.9	58.63	6.54	2.07	7.64	11.03	0	0	Sinopharm – 14.09	18/01/2021	35443	159957
Venezuela	28436000	32.3	n/d	16244	50.2	0	0	0	0	Sputnik V		Abdala	Sinopharm Soberana 02 Soberana Plus	17/02/2021	4732	11512
Gambia	2417000	232	n/d	5111	14.0	0	0	Johnson & Johnson	Oxford / AstraZeneca	0	0	0	Sinopharm	09/03/2021	1793	3318
Ghana	31073000	133.7	77	5328	19.7	0	0	0	100		0	0	0	01/03/2021	2520	2808
Guinea-Bissau	1968000	68.3	68	5139	17.4	0	0	0	Oxford / AstraZeneca	0	0	0	Sinopharm	12/05/2021	1777	3362
Germany	83784000	239.6	1536	308600	78.5	73.72	17.11	2.06	7.04	0	0	Novavax – 0.07	0	27/12/2020	25706	282894
China Hong Kong	7497000	7082.1	5981	161440	83.79	58.99	0	0	0	0	0	0	Sinovac – 41.01	22/02/2021	NA	161440
Greenland	57000	0.1	n/d	213459	50.2	0	100	0	0	0	0	0	0	27/01/2021	531	212928
Denmark	5792000	136	11080	538246	84.1	84.27	14.07	0.38	1.28	0	0	0	0	18/12/2020	21049	517197
Democratic Republic of the Congo	89561000	38.3	11	978	0.6	0	0	0	100	0	0	0	0	18/04/2021	292	686
Zambia	18384000	24	183	17958	16	0	0	Johnson & Johnson	Oxford / AstraZeneca	0	0	0	Sinopharm	14/04/2021	4515	13443
Zimbabwe	14863000	37.9	152	16537	30.1	0	0	0	Oxford / AstraZeneca	Sputnik V	0	0	Sinovac Sinopharm	18/02/2021	2170	14367
Israel	8656000	393.7	5459	449351	66.6	100		0	0	0	0	0	0	19/12/2020	39477	409874
India	1380004000	459.6	604	31708	63.9	0	0	0	79.97	0	0	Corbevax – 3.25	Covaxin – 16.78	15/01/2021	7429	24279
Iran	83993000	50.9	607	86766	68.8	0	0	0	Oxford / AstraZeneca	Sputnik V	0	Razi Cov Pars SpikoGen	COVIran Barekat Covaxin FAKHRAVAC Sinopharm Soberana 02	08/02/2021	16560	70206
Ireland	4938000	70.9	2436	311400	63.2	74.83	12.26	2.14	10.76	0	0	Novavax – <0.01	0	28/12/2020	16957	294443
Country	Population	Population density. per km^2	PCR testing coverage, ‱	Reported COVID-19 cases, per 1 million population	Vaccination coverage. %	mRNA Vaccines (V1), %		Vector Vaccines (V2), %				Protein, Peptide Vaccines (V3), %	Whole-virion, Inactivated Vaccines (V4), %	Beginning of national vaccination company	Reported COVID-19 cases, per 1 million population before beginning of vaccination	Reported COVID-19 cases, Reported COVID-19 cases, per 1 million population after beginning of vaccination
						Pfizer / BioNTech	Moderna	Johnson & Johnson	Oxford / AstraZeneca, Covishield	Sputnik V (Gam-COVID-Vac)	CanSino					
Iceland	341000	3.4	1951	511100	86.3	92.09	7.81	0.1	<0.01	0	0	0	0	30/12/2020	15400	495700

Spain	46755000	93.7	1964	256000	87.2	64.97	23.46	1.95	9.62	0	0	0	0	04/01/2021	42442	213558
Italy	60462000	205.9	3597	281400	79.0	65.31	24.72	1.1	8.85	0	0	Novavax – 0.03	0	27/12/2020	34691	246709
Kazakhstan	18777000	6.9	549	74690	60.4	0	0	0	0	Sputnik V	0	0	QazVac Sinopharm/Beijing	31/01/2021	12092	62598
Canada	37742000	4.1	1613	100800	86.7	68.36	28.26	0.03	3.35	0	0	Novavax – <0.01	0	14/12/2020	12017	88783
Cyprus	1207000	129.7	32860	552200	56.5	71.98	11.43	1.79	14.79	0	0	Novavax – <0.01	0	06/01/2021	29284	522916
Kyrgyzstan	6524000	33.5	NA	30763	58.3	0	0	0	0	16.6	0	EpiVacCorona – 5.5	Sinopharm – 77.9	28/03/2021	954	29809
Kiribati	119000	145.2	NA	3098	50.8	0	0	0	100	0	0	0	0	01/06/2021	0	3098
China	1439324000	152.7	6380	617	88	0	0	0	0	0	CanSino	0	IMBCAMS KCONVAC Sinovac Sinopharm	10/06/2021	81	536
Union of the Comoros	870000	457.2	NA	9287	34.6	0	0	0	Oxford / AstraZeneca	0	0	0	Covaxin Sinopharm	21/04/2021	4567	4720
Cuba	11327000	106.5	NA	98577	87.7	0	0	0	0	0	0	Abdala	Soberana 02 Soberana Plus	12/05/2021	10640	87937
Curaçao	164000	368.1	134	274301	64	100		0	0	0	0	0	0	29/05/2021	64188	210113
Latvia	1886000	30.7	3851	432900	20.1	56.04	24.72	10.16	9.07	0	0	Novavax – 0.01	Sinovac – <0.01 Sinopharm – <0.01	04/12/2020	11516	421384
Lesotho	2142000	70	209	16593	34.3	0	0	Johnson & Johnson	Oxford / AstraZeneca	0	0	0	Sinopharm	09/03/2021	4565	12028
Liberia	5058000	51.3	NA	1666	28.4	0	0	0	100	0	0	0	0	12/05/2021	403	1263
Lithuania	2722000	44	3227	379500	56.8	74.04	7.35	6.61	12	0	0	0	0	27/12/2020	48806	330694
Liechtenstein	38000	237.6	NA	446600	70.7	31.36	68.25	0.39	0	0	0	0	0	21/12/2020	48100	398500
Luxembourg	626000	237.7	6692	396800	80.1	60.71	26.63	3.57	9.05	0	0	Novavax – 0.03	0	28/12/2020	59269	337531
Mauritius	1272000	625.5	1007	177508	76.1	0	0	0	Oxford / AstraZeneca	0	0	0	Covaxin Sinopharm	25/01/2021	437	177072
Mauritania	4650000	4.4	201	14482	23.3	0	0	0	Oxford / AstraZeneca	0	0	0	Sinopharm	26/03/2021	3747	10735
Madagascar	27691000	46.4	15	2450	4.2	0	0	Johnson & Johnson	Oxford / AstraZeneca	0	0	0	Sinopharm	12/05/2021	1334	1116
Malawi	19130000	197.6	29	4492	6	0	0	100		0	0	0	0	17/03/2021	1612	2880
Mali	20251000	16.1	33	1555	5.3	0	0	0	100	0	0	0	0	30/03/2021	439	1116
Malta	525285	1376.2	3693	188900	96.1	58.29	20.66	2.62	18.44	0	0	0	0	17/01/2021	30073	158827
Mozambique	31255000	38.6	41	7503	44.8	0	0	Johnson & Johnson	Oxford / AstraZeneca	0	0	0	Sinopharm	07/03/2021	1885	5618
Monaco	39000	26152.3	NA	316554	65.4	100	0	0	0	0	0	0	0	04/03/2021	49903	266651
Montserrat	5000	49.9	NA	195105	36.6	0	0	0	100	0	0	0	0	08/02/2021	3852	191253
Myanmar	54410000	82.7	148	11286	44.7	0	0	0	Oxford / AstraZeneca	0	0	0	Sinopharm	26/01/2021	2560	8726
Nepal	29137000	199.6	231	32600	74.6	1.47	13.95	8.31	29.7	0	0	0	Sinopharm – 46.58	01/02/2021	8880	23720
Niger	24207000	18.4	10	405	6.4	0	0	0	Oxford / AstraZeneca	0	0	0	Sinopharm	28/03/2021	190	215

Country	Population	Population density. per km^2	PCR testing coverage, ‱	Reported COVID-19 cases, per 1 million population	Vaccination coverage. %	mRNA Vaccines (V1), %		Vector Vaccines (V2), %				Protein, Peptide Vaccines (V3), %	Whole-virion, Inactivated Vaccines (V4), %	Beginning of national vaccination company	Reported COVID-19 cases, per 1 million population before beginning of vaccination	Reported COVID-19 cases, Reported COVID-19 cases, per 1 million population after beginning of vaccination
						Pfizer / BioNTech	Moderna	Johnson & Johnson	Oxford / AstraZeneca, Covishield	Sputnik V (Gam-COVID-Vac)	CanSino					
Nigeria	206140000	220.7	24	1242	8.2	0	0	0	100	0	0	0	0	04/03/2021	721	521
Netherlands	17135000	507	1781	467829	73.0	67.04	23.4	2.23	7.32	0	0	Novavax – <0.01	0	08/01/2021	47207	420622
New Caledonia	285000	15.5	NA	218462	66.2	100	0	0	0	0	0	0	0	02/02/2021	162	218300
Norway	5421000	14.7	2031	266730	77.4	78.56	20.07	0.06	1.31	0	0	0	0	02/12/2020	6815	259915
Cook Islands	18000	73.1	NA	237	84.1	100	0	0	0	0	0	0	0	25/05/2021	0	237
Turks and Caicos Islands	39000	40.2	NA	147381	76.1	100	0	0	0	0	0	0	0	10/01/2021	21104	126277
Papua New Guinea	8947000	19.4	NA	4970	3	0	0	0	100	0	0	0	0	30/03/2021	591	4379
Peru	32972000	25.4	875	111100	87	60.6	0.8	0	10.7	0	0	0	Sinopharm – 27.9	09/02/2021	34670	76430
Poland	37847000	123.7	972	156400	58.4	76.94	7.22	5.35	10.47	0	0	Novavax – 0.02	0	28/12/2020	31890	124510
Portugal	10197000	111.7	4106	390700	85.6	68.42	16.29	5.1	10.15	0	0	Novavax – <0.01	Covaxin – <0.01 Sinovac – 0.02 Sinopharm – 0.01	01/01/2021	36756	353944
Russia	145934000	8.9	2001	122871	51.1	0	0	0	0	95.5%	0	EpiVacCorona – 3.3%	CoviVac – 1.2%	15/12/2020	18895	103976
Romania	19238000	84.1	NA	149500	41.5	76.73	6	12.22	5.06	0	0	0	0	27/12/2020	31220	118280
Samoa	198000	69.6	NA	106014	94.2	0	0	0	100	0	0	0	0	04/05/2021	5	106009
Sao Tome and Principe	219000	224	13	29646	39.7	0	0	0	100	0	0	0	0	15/03/2021	9138	20508
Senegal	16744000	84.6	64	5099	81.7	0	0	Johnson & Johnson	Oxford / AstraZeneca	0	0	0	Sinopharm	22/02/2021	1911	3188
Seychelles	98000	212.5	NA	447928	81.7	0	0	0	Oxford / AstraZeneca	Sputnik V	0	0	Sinopharm	09/01/2021	4685	443243
Slovakia	5460000	113.5	9380	466345	51.0	74.62	9.83	2.7	12.29	0.55	0	Novavax – 0.02	0	03/01/2021	33330	433015
Slovenia	2079000	103.2	2491	482193	59.0	76.38	8.03	4.61	10.98	0	0	Novavax – <0.01	0	27/12/2020	54443	427750
United States	331003000	36	2659	247232	59.2	59.03	37.75	3.22	0	0	0	0	0	13/12/2020	47975	199257
Sierra Leone	7977000	108.2	52	912	68.1	0	0	0	Oxford / AstraZeneca	0	0	0	Sinopharm	14/03/2021	457	455
Tajikistan	9538000	66.6	50	1787	16.5	9.7	24.3	0	20.2	0	0	0	Sinovac – 45.8	07/05/2021	1378	409
Togo	8279000	148.6	87	4285	57.8	0	0	0	100	0	0	0	0	09/03/2021	868	3417
Uruguay	3474000	19.8	1746	265303	18.8	43.03	0	0	1.55	0	0	0	Sinovac – 55.42	27/02/2021	16272	249031
Faroe islands	49000	34.9	NA	664914	83.7	100		0	0	0	0	0	0	29/01/2021	12369	652545
Finland	5541000	18.2	1954	193500	78.8	79.21	16.1	0	4.68	0	0	0	0	03/01/2021	7188	186312
Falkland Islands	3000	0.3	NA	296237	62.5	0	0	0	100	0	0	0	0	07/02/2021	12628	283609

France	65274000	118.9	4034	422000	79.1	77.39	16.4	0.75	5.44	0	0	Novavax – 0.01	0	27/12/2020	36181	385819
Croatia	4105000	73.8	1190	277200	53.5	75.47	9.78	3.87	10.86	0	0	Novavax – 0.02	0	30/12/2020	51718	225482
Central African Republic	4830000	7.6	19	2665	20.5	0	0	0	Oxford / AstraZeneca	0	0	0	Covaxin	12/05/2021	1908	757
Chad	16426000	12.7	13	457	12.7	0	0	0	0	0	0	0	Sinopharm – 100	10//01/2021	143	314
Czech Republic	10709000	138.4	5072	399774	65.1	83.51	9.07	2.34	5.04	0	0	Novavax – 0.04	Covaxin – <0.01 Sinovac – <0.01 Sinopharm – <0.01	27/12/2020	64328	335446
Chile	19116000	25.5	1956	187900	96.9	23.26	1.6	0	0	0	0	0	Sinovac – 75.14	24/12/20	30265	157635
Switzerland	8655000	217.4	2404	421098	71.9	37.21	62.41	0.39	0	0	0	0	0	21/12/2020	47307	373791
Sweden	10099000	24.5	1802	242400	77.1	74.77	18.94	0	6.26	0	0	Novavax – 0.03	0	03/01/2021	433596	198804
Ecuador	17643000	70	154	49900	86.4	28.59	0	0	16.75	0	1.79	0	Sinovac – 52.87	20/01/2021	12920	36980
Equatorial Guinea	1403000	48.3	331	11713	15.3	0	0	0	0	0	0	0	Sinovac + Sinopharm = 100	16/03/2021	3942	7771
Estonia	1327000	31.3	2544	432100	64.0	73.28	11.43	3.77	11.48	0	0	Novavax – 0.04	0	27/12/2020	23400	408700
Ethiopia	114964000	112.1	41	7512	18.5	0	0	Johnson & Johnson	Oxford / AstraZeneca	0	0	0	Covaxin Sinovac Sinopharm	08/04/2021	1813	5699
South Africa	59309000	48.3	413	65900	96.9	75.57	0	24.43	0	0	0	0	0	16/02/2021	24926	40974
South Korea	51269000	526.8	1891	342300	87.7	62.4	19.72	1.23	16.26	0	0	Novavax – 0.4	0	26/02/2021	1730	340570
South Sudan	11194000	18.1	35	1375	5.6	0	0	100		0	0	0	0	05/04/2021	942	433
Japan	126476000	348	382	69500	81.2	77.16	22.8	0	0.04	0	0	0	0	17/02/2021	3404	66096

Notes:
* Vaccines used were divided into four groups. V1 – mRNA vaccines: mRNA-1273 (Moderna), BNT162b2 (Pfizer/BioNTech). V2 – vector vaccines: Johnson & Johnson, Oxford/AstraZeneca (Covishield), Gam-COVID-Vac (Sputnik V), CanSino. V3 – protein and peptide vaccines: EpiVacCorona, Novavax. V4 – whole-virion vaccines: Sinovac, Sinopharm, Soberana, COVIran Barekat, Covaxin, FAKHRAVAC, Corbevax, QazVac, IMBCAMS, KCONVAC, CoviVac.
Sources:
Population number and density were taken from Country Meters Info (https://countrymeters.info), accessed 05/05/2002. Prevalence per 1 million population, COVID-19 vaccination coverage, and vaccine ratios were taken from: Our World in Data (https://ourworldindata.org/), accessed 13/05/2022; Gogov Statistics (https://gogov.ru/articles/covid-19), accessed 13-15/05/2022; Coronavirus Monitor Info (https://coronavirus-monitor.info/), accessed 10-20/05/2022; the World Health Organization (https://covid19.who.int/), accessed 10-20/05/2022; and the Pan American Health Organization (https://www.paho.org/), accessed 15-20/05/2022. See also link (https://ourworldindata.org/grapher/covid-vaccine-doses-by-manufacturer?country). When necessary, official information was taken from various government websites: UK Health Security Agency (https://assets.publishing.service.gov.uk/government/uploads/system/uploads/attachment_data/file/1075115/COVID-19_vaccine_surveillance_report_12_May_2022_week_19.pdf). # Bhutan: 1st vaccination (94% of adults) – Oxford/AstraZeneca; 2nd vaccination (90.2%) – Moderna

mRNA-1273 (Tamang S.T., Dorji T.; 'COVID-19 vaccinations in Bhutan – Mix-and-Match to Boosters: An experience'; Vaccine. 2022; 40(23): 3089–3092. doi: 10.1016/j.vaccine.2022.04.059).

Table S2. Data set adjusted for population and vaccination coverage (n=53).

Country	Population	Population density, per km^2	PCR testing coverage, ‱	Reported COVID-19 cases, per 1 million population	Vaccination coverage, %	mRNA Vaccines (V1), %			Vector Vaccines (V2), %					Protein, Peptide Vaccines (V3), %		Whole-virion, Inactivated Vaccines (V4), %		Share of vaccine usage, %		Beginning of national vaccination company	Reported COVID-19 cases, per 1 million population before beginning of vaccination	Reported COVID-19 cases, Reported COVID-19 cases, per 1 million population after beginning of vaccination
						Pfizer / BioNTech	Moderna	V1 total	Johnson & Johnson	Oxford / AstraZeneca, Covishield	Sputnik V (Gam-COVID-Vac)	CanSino	V2 total		V3 total		V4 total	V1	Vnmg (V2+ V3+ V4)			
Australia	25500000	3.3	2755	266595	83.29	55.37	0	55.37	0	44.63	0	0	44.63	0	0	0	0	55.37	44.63	21/02/2021	1105	265490
Austria	9006000	108.7	20551	480347	75.8	80.65	8.68	89.33	1.99	8.63	0	0	10.62	Novavax – 0.05	0.05	0	0	89.33	10.67	08/01/2021	41364	438983
Argentina	46403000	16.4	791	202500	86.1	17.16	7.62	24.78	0	26.08	20.19	0.78	47.05	0	0	Sinopharm – 28.16	28.16	24.78	75.22	29/12/2020	36361	166139
Belarus	9449000	46.6	1400	104411	63.8	0	0	0	0	0	57.4	0	57.4	EpiVacCorona – 0.3	0.3	Sinovac – 0.6 Sinopharm – 32.3 CoviVac – 0.5	33.4	0	100	28/12/2020	19778	84633
Belgium	11590000	381.1	2920	357100	81.23	70.15	16.92	87.07	1.69	11.24	0	0	12.93	Novavax – <0.01	0	0	0	87.07	12.93	28/12/2020	54940	302160
Bhutan [#]	772000	20	2170	80378	90.46	0	50	50	0	50	0	0	50	0	0	0	0	50	50	26/03/2021	1113	79260
Great Britain	67886000	279.1	7348	331600	79.0	48.7	3	51.7	0	48.3	0	0	48.3	0	0	0	0	51.7	48.3	10/01/2021	46584	285076
Hungary	9660000	107	1136	195400	62.9	58.63	6.54	65.17	2.07	7.64	11.03	0	20.74	0	0	Sinopharm – 14.09	14.09	65.17	34.83	18/01/2021	35443	159957
Venezuela	28436000	32.3	n/d	16244	50.2	0	0	0	0	0	Sputnik V	0	NA	Abdala	n/d	Sinopharm Soberana 02 Soberana Plus	NA	0	100	17/02/2021	4732	11512
Germany	83784000	239.6	1536	308600	78.5	73.72	17.11	90.83	2.06	7.04	0	0	9.1	Novavax – 0.07	0.07	0	0	90.83	9.17	27/12/2020	25706	282894
China Hong Kong	7497000	7082.1	5981	161440	83.79	58.99	0	58.99	0	0	0	0	0	0	0	Sinovac – 41.01	41.01	58.99	41.01	22/02/2021	NA	161440
Denmark	5792000	136	11080	538246	84.1	84.27	14.07	98.34	0.38	1.28	0	0	1.66	0	0	0	0	98.34	1.66	18/12/2020	21049	517197
Israel	8656000	393.7	5459	449351	66.6	100		100	0	0	0	0	0	0	0	0	0	100	0	19/12/2020	39477	409874
India	1380004000	459.6	604	31708	63.9	0	0	0	0	79.97	0	0	79.97	0	Corbevax -3.25	Covaxin – 16.78	20.03	0	100	15/01/2021	7429	24279
Country	Population	Population density, per km^2	PCR testing coverage, ‱	Reported COVID-19 cases, per 1 million population	Vaccination coverage, %	mRNA Vaccines (V1), %			Vector Vaccines (V2), %					Protein, Peptide Vaccines (V3), %		Whole-virion, Inactivated Vaccines (V4), %		Share of vaccine usage, %		Beginning of national vaccination company	Reported COVID-19 cases, per 1 million population before beginning of vaccination	Reported COVID-19 cases, Reported COVID-19 cases, per 1 million population after beginning of vaccination
						Pfizer / BioNTech	Moderna	V1 total	Johnson & Johnson	Oxford / AstraZeneca,	Sputnik V (Gam-COVID-Vac)	CanSino	V2 total		V3 total		V4 total	V1	Vnmg (V2+ V3+ V4)			

										Covishield												
Iran	83993000	50.9	607	86766	68.8	0	0	0	0	Oxford/AstraZeneca	Sputnik V	0	n/d	Razi Cov Pars Spiko Gen	n/d	COVIran Barekat Covaxin FAKHRAVAC Sinopharm Soberana 02	n/d	0	100	08/02/2021	16560	70206
Ireland	4938000	70.9	2436	311400	63.2	74.83	12.26	87.09	2.14	10.76	0	0	12.9	Novavax – <0.01	0.01	0	0	87.09	12.91	28/12/2020	16950	294443
Spain	46755000	93.7	1964	256000	87.2	64.97	23.46	88.43	1.95	9.62	0	0	11.57	0	0	0	0	88.43	11.57	04/01/2021	42442	213558
Italy	60462000	205.9	3597	281400	79.0	65.31	24.72	90.03	1.1	8.85	0	0	9.95	Novavax – 0.03	0.03	0	0	90.03	9.97	27/12/2020	34691	246709
Kazakhstan	18777000	6.9	549	74690	60.4	0	0	9.7	0	0	Sputnik V	0	NA	0	0	QazVac Sinopharm/Beijing	NA	9.7	90.3	31/01/2021	12092	62598
Canada	37742000	4.1	1613	100800	86.7	68.36	28.26	96.62	0.03	3.35	0	0	3.38	Novavax – <0.01	0	0	0	96.62	3.38	14/12/2020	12017	88783
Cyprus	1207000	129.7	32860	552200	56.5	71.98	11.43	83.41	1.79	14.79	0	0	16.58	Novavax – <0.01	0.01	0	0	83.41	16.59	06/01/2021	29284	522916
Kyrgyzstan	6524000	33.5	n/d	30763	58.3	0	0	0	0	0	16.6	0	16.6	EpiVacCorona – 5.5	5.5	Sinopharm – 77.9	77.9	0	100	28/03/2021	954	29809
China	1439324000	152.7	6380	617	88	0	0	0	0	0	0	CanSino	n/d	0	0	IMBCAMS KCONVAC Sinovac Sinopharm	NA	0	100	10/06/2021	81	536
Cuba	11327000	106.5	n/d	98577	87.7	0	0	0	0	0	0	0	0	Abdala	n/d	Soberana 02 Soberana Plus	NA	0	100	12/05/2021	10640	87937
Lithuania	2722000	44	3227	379500	56.8	74.04	7.35	81.39	6.61	12	0	0	18.61	0	0	0	0	81.39	18.61	27/12/2020	48806	330694
Luxembourg	626000	237.7	6692	396800	80.1	60.71	26.63	87.34	3.57	9.05	0	0	12.62	Novavax – 0.03	0.03	0	0	87.34	12.66	28/12/2020	59269	337531
Mauritius	1272000	625.5	1007	177508	76.1	0	0	0	0	Oxford/AstraZeneca	0	0	n/d	0	0	Covaxin Sinopharm	n/d	0	100	25/01/2021	437	177071
Malta	525285	1376.2	3693	188900	96.1	58.29	20.66	78.95	2.62	18.44	0	0	21.05	0	0	0	0	78.95	21.05	17/01/2021	30073	158827
Nepal	29137000	199.6	231	32600	74.6	1.47	13.95	15.42	8.31	29.7	0	0	38.01	0	0	Sinopharm – 46.58	46.58	15.42	84.58	01/02/2021	8880	23720
Netherlands	17135000	507	1781	467829	73.0	67.04	23.4	90.44	2.23	7.32	0	0	9.55	Novavax – <0.01	0.01	0	0	90.44	9.56	08/01/2021	47207	420622
Norway	5421000	14.7	2031	266730	77.4	78.56	20.07	98.63	0.06	1.31	0	0	1.37	0	0	0	0	98.63	1.37	02/12/2020	6815	259915
Peru	32972000	25.4	875	111100	87	60.6	0.8	61.4	0	10.7	0	0	10.7	0	0	Sinopharm – 27.9	27.9	61.4	38.6	09/02/2021	34670	76430

Country	Population	Population density, per km^2	PCR testing coverage, ‱	Reported COVID-19 cases, per 1 million population	Vaccination coverage, %	mRNA Vaccines (V1), %			Vector Vaccines (V2), %					Protein, Peptide Vaccines (V3), %		Whole-virion, Inactivated Vaccines (V4), %		Share of vaccine usage, %		Beginning of national vaccination company	Reported COVID-19 cases, per 1 million population before beginning of vaccination	Reported COVID-19 cases, Reported COVID-19 cases, per 1 million population after beginning of vaccination
						Pfizer / BioNTech	Moderna	V1 total	Johnson & Johnson	Oxford / AstraZeneca, Covishield	Sputnik V (Gam-COVID-Vac)	CanSino	V2 total		V3 total		V4 total	V1	Vnmg (V2+ V3+ V4)			
Poland	37847000	123.7	972	156400	58.4	76.94	7.22	84.16	5.35	10.47	0	0	15.82	Novavax – 0.02	0.02	0	0	84.16	15.84	28/12/2020	31890	124510
Portugal	10197000	111.7	4106	390700	85.6	68.42	16.29	84.71	5.1	10.15	0	0	15.25	Novavax – <0.01	0	Covaxin – <0.01 Sinovac – 0.02 Sinopharm – 0.01	0.04	84.71	15.29	01/01/2021	36756	353944
Russia	145934000	8.9	2001	122871	51.1	0	0	0	0	0	95.5%	0	95.5	EpiVacCorona – 3.3%	3.3	CoviVac – 1.2%	1.2	0	100	15/12/2020	18895	103976
Senegal	16744000	84.6	64	5099	81.7	0	0	0	Johnson & Johnson	Oxford/AstraZeneca	0	0	n/d	0	0	Sinopharm	n/d	0	100	22/02/2021	1911	3188
Slovakia	5460000	113.5	9380	466345	51.0	74.62	9.83	84.45	2.7	12.29	0.55	0	15.54	Novavax – 0.02	0.02	0	0	84.45	15.55	03/01/2021	33330	433015
Slovenia	2079000	103.2	2491	482193	59.0	76.38	8.03	84.41	4.61	10.98	0	0	15.59	Novavax – <0.01	0	0	0	84.41	15.59	27/12/2020	54443	427750
United States	331003000	36	2659	247232	59.2	59.03	37.75	96.78	3.22	0	0	0	3.22	0	0	0	0	96.78	3.22	13/12/2020	47975	199257
Sierra Leone	7977000	108.2	52	912	68.1	0	0	0	0	Oxford/AstraZeneca	0	0	n/d	0	0	Sinopharm	n/d	0	100	14/03/2021	457	455
Togo	8279000	148.6	87	4285	57.8	0	0	0	0	100	0	0	100	0	0	0	0	0	100	09/03/2021	868	3417
Finland	5541000	18.2	1954	193500	78.8	79.21	16.1	95.31	0	4.68	0	0	4.68	0	0	0	0	95.31	4.69	03/01/2021	7188	186312
France	65274000	118.9	4034	422000	79.1	77.39	16.4	93.79	0.75	5.44	0	0	6.19	Novavax – 0.01	0.01	0	0	93.79	6.21	27/12/2020	36181	385819
Croatia	4105000	73.8	1190	277200	53.5	75.47	9.78	85.27	3.87	10.86	0	0	14.73	Novavax – 0.02	0.02	0	0	85.27	14.73	30/12/2020	51718	225482
Czech Republic	10709000	138.4	5072	399774	65.1	83.51	9.07	92.58	2.34	5.04	0	0	7.38	Novavax – 0.04	0.04	Covaxin – <0.01 Sinovac – <0.01 Sinopharm – <0.01	0	92.58	7.42	27/12/2020	64328	335446
Chile	19116000	25.5	1956	187900	96.9	23.26	1.6	24.86	0	0	0	0	0	0	0	Sinovac – 75.14	75.14	24.86	75.14	24/12/2020	30265	157635
SwitzerlaNA	8655000	217.4	2404	421098	71.9	37.21	62.41	100	0.39	0	0	0	0.39	0	0	0	0	100	0	21/12/2020	47307	373791
Sweden	10099000	24.5	1802	242400	77.1	74.77	18.94	93.71	0	6.26	0	0	6.26	Novavax – 0.03	0.03	0	0	93.71	6.29	03/01/2021	43596	198804
Ecuador	17643000	70	154	49900	86.4	28.59	0	28.59	0	16.75	0	1.79	18.54	0	0	Sinovac – 52.87	52.87	28.59	71.41	20/01/2021	12920	36980
Estonia	1327000	31.3	2544	432100	64.0	73.28	11.43	84.71	3.77	11.48	0	0	15.25	Novavax – 0.04	0.04	0	0	84.71	15.29	27/12/2020	23400	408700

South Africa	59309000	48.3	413	65900	96.9	75.57	0	75.57	24.43	0	0	0	24.43	0	0	0	0	75.57	24.43	16/02/2021	24926	40974
South Korea	51269000	526.8	1891	342300	87.7	62.4	19.72	82.12	1.23	16.26	0	0	17.49	Novavax – 0.4	0.4	0	0	82.12	17.88	26/02/2021	1730	340570
Japan	126476000	348	382	69500	81.2	77.16	22.8	100	0	0.04	0	0	0.04	0	0	0	0	100	0	17/02/2021	3404	66096

Notes: Please see notes for Table 1S.

Table S3. Analysis of prevalence in the compared subgroups (of Fig. 10B).

Pairwise group comparison	U	Z	P
V1>Vnmg (subgroup 1) ↔ V1≈Vnmg (subgroup 2)	37.00000	2.100656	0.035672
V1≈Vnmg (subgroup 2) ↔ V1<Vnmg (subgroup 3)	15.00000	2.485497	0.009867
V1>Vnmg (subgroup 1) ↔ V1<Vnmg (subgroup 3)	26.00000	4.951293	0.000001

Abbreviations: U – Mann-Whitney test value; Z – z-score; P – statistical significance level (differences).

Table S4. Discriminant function analysis and classification error matrix.

Discriminant Function Analysis: Wilks' Lambda: 0.18634; approx. F (4.92) = 30.282; *p*<0.00001

	Wilks' - Lambda	Partial - Lambda	F-remove - (2.46)	p-value	Toler.	1-Toler. - (R-Sqr.)
PCR testing coverage	0.427103	0.436283	29.71807	0.0000001	0.983416	0.016584
prevalence per 1 million people	0.302642	0.615704	14.35563	0.000014	0.983416	0.016584

Classification error matrix N = 50; (3 groups). Rows – observed classification; columns – predicted classification

	Percent Correct	Group 1 *p*=0.26	Group 2 *p*=0.66	Group 3 *p*=0.08
group 1 (n=13)	84.61539	11	2	0
group 2 (n=33)	87.87878	4	29	0
group 3 (n=4)	75.00000	0	1	3
TOTAL	86.00000	15	32	3

Table S5. Analysis of prevalence in the compared subgroups (of Fig. 10B).

Pairwise group comparison	U	Z	P
V1>Vnmg (subgroup 1) ↔ V1≈Vnmg (subgroup 2)	21.00000	2.159460	0.027391

V1≈Vnmg (subgroup 2) ↔ V1<Vnmg (subgroup 3)	6.000000	1.253359	0.210076
V1>Vnmg (subgroup 1) ↔ V1<Vnmg (subgroup 3)	4.000000	-3.17921	0.000244

Abbreviations: U – Mann-Whitney test value; Z – z-score; P – statistical significance level (differences).
